# Pool Toes: A Case Report

**DOI:** 10.1159/000529079

**Published:** 2023-02-03

**Authors:** Mohammad Munshi, Luca Borradori, Nikhil Yawalkar, Kristine Heidemeyer

**Affiliations:** Department of Dermatology, Inselspital - Bern University Hospital, University of Bern, Bern, Switzerland

**Keywords:** Pool, Dermatitis, Palm, Aquatic, Dermatoses, Feet, Fresh, Pedal, Swimming, Toes, Water

## Abstract

Pool toes, a sport-related dermatosis, are caused by mechanical friction and water exposure, resulting in a special variant of irritant contact dermatitis. It is common in children, often misdiagnosed, and rarely reported. Here we report a case of a 7-year-old girl who developed this unusual type of frictional dermatitis; a pool toes diagnosis has been made. With topical corticosteroids, favorable results have been achieved. The recovery and healing process will be facilitated if one is aware of the underlying causes of such dermatitis and ceases the triggering factors.

## Introduction

Cohen first described pool toes in 2005 as a sport-related dermatosis that mainly affected adolescents and children [1]. This benign condition is thought to result from contact with the sometimes rough, non-slip outdoor surface of the pool deck or the pool floor. Since both hands and feet are often affected, the name pool palms and feet has been proposed [1–3]. The lesions usually resolve within a few days, and recurrence after re-exposition is possible.

## Case Presentation

**Fig. 1.**
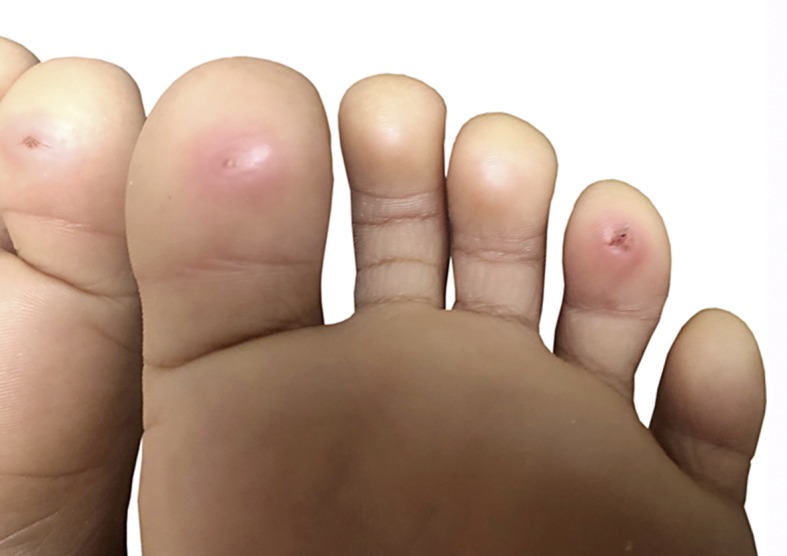
Symmetrical erythematous well-demarcated macules involving the pulps of first and 4th toes.

**Fig. 2.**
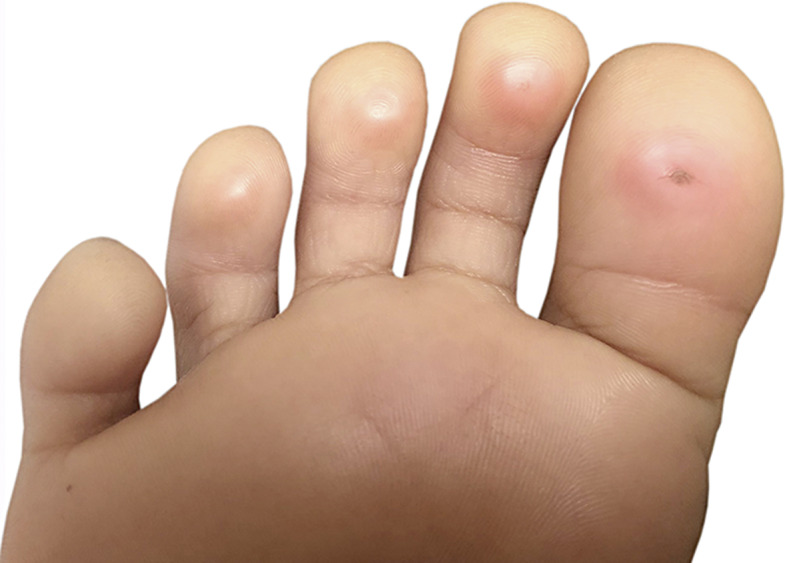
Erythematous macules with central minute erosions affecting the pulps of the toes I and IV.

A 7-year-old girl presented with painful burning macules at the tip of her toes with small vesicles, which developed after repeatedly using a swimming pool during the summer (Fig. 1, 2). An examination revealed symmetrically distributed erythematous macules in combination with superficial erosions on the 1st to 4th toes. Based on the patient’s history and physical examination, the diagnosis of pool toes was made. No further exams were performed. The patient was given mometasone cream to apply once daily on the affected feet and was instructed to temporarily stop swimming. At the follow-up visit after 2 weeks, all lesions had resolved completely. The CARE Checklist has been completed by the authors for this case report, attached as online supplementary material (for all online suppl. material, see www.karger.com/doi/10.1159/000529079).

## Discussion

Although pool toes and palms are rarely described in the literature, they are likely to occur quite frequently. It is a type of friction dermatitis that usually affects fingertips, toes, palms, and heels. Its development is most likely favored by the more fragile skin in children in combination with prolonged water exposure, leading to over-hydration of the epidermis and mechanical friction. The latter is caused by the sometimes rough anti-slip surface of the swimming pool [3–5]. Playing and running in the swimming pool and/or on the pool deck in this young age group may cause irritant contact dermatitis due to the repeated rubbing of the extremities on rough surfaces [4].

Several conditions must be differentiated and excluded, such as allergic contact dermatitis, digital pulpitis, juvenile plantar dermatosis (JPD), and, recently, COVID toes [6]. Allergic contact dermatitis is characterized by itching rather than pain and does not depend on the frequency of contact. Digital pulpitis is an irritant chemical and mechanical dermatitis commonly seen in adults. JPD is characterized by scaling, peeling, and fissuring at the base of the great toe, which can spread to the heel and ventral foot. JPD is much more common in children with atopic dermatitis when it is not seasonal. Histologically, dermal infiltration has been observed in the acrosyringium [7]. Finally, during the COVID-19 pandemic, several children and teenagers have been described with so-called COVID toes, which may occur in combination with flu-like symptoms and fever. One or many toes may be affected and show swelling with a red, purple, or blue skin discoloration with variable papules and blistering. The lesions almost invariably have a favorable prognosis [6].

## Conclusion

Although cases of pool toes and pool palms have been previously reported, knowledge of this benign condition remains scarce among dermatologists, pediatricians, and other health care providers. The diagnosis can be easily made based on a good clinical history and clinical examination. The use of topical corticosteroids of medium strength combined with a reduction in recreational swimming pool activities will invariably resolve the lesions.

## Statement of Ethics

All procedures adopted in the present study respect the ethical standards of the World Medical Association Declaration of Helsinki. Ethical approval was not required for this study, following local guidelines. Written informed consent was obtained from the patient’s father to publish this case report and any accompanying images.

## Conflict of Interest Statement

The authors have no conflicts of interest to declare.

## Funding Sources

The authors received no funding for any aspect of this manuscript.

## Author Contributions

Mohammad Munshi: acquisition of data and writing of the paper. Luca Borradori and Kristine Heidemeyer: critically revising the work. Nikhil Yawalkar: acquisition of data and critically revising the work. All authors have given the final approval of the version to be published and agree to be accountable for all aspects of the work in ensuring that questions related to the accuracy or integrity of any part of the work are appropriately investigated and resolved.

## Data Availability

All data generated or analyzed during this study are included in this article and its online supplementary material. Further inquiries can be directed to the corresponding author.
